# A Case of Exacerbation of Subclinical Hyperthyroidism after First Administration of BNT162b2 mRNA COVID-19 Vaccine

**DOI:** 10.3390/vaccines9101108

**Published:** 2021-09-29

**Authors:** Kana Yamamoto, Takahisa Mashiba, Keisuke Takano, Toshihiko Suzuki, Masahiro Kami, Morihito Takita, Eiji Kusumi, Yasuhiro Mizuno, Tamae Hamaki

**Affiliations:** 1 Department of Reproductive, Developmental and Aging Sciences, Graduate School of Medicine, University of Tokyo, 4-6-1 Shirokanedai, Minato, Tokyo 108-0071, Japan; 2 Department of Internal Medicine, Navitas Clinic Tachikawa, 3-1-1 Shibasakicho 4th Floor of Ecute Tachikawa, Tachikawa, Tokyo 190-0023, Japan; takita-ygc@umin.net (M.T.); eiji.kusumi@gmail.com (E.K.); 3 Department of General Internal Medicine, Tokyo Bay Urayasu Ichikawa Medical Center, 3-4-32 Todaijima, Urayasu, Chiba 279-0001, Japan; takamassy99@gmail.com; 4 Department of Endocrinology, Tokyo Bay Urayasu Ichikawa Medical Center, 3-4-32 Todaijima, Urayasu, Chiba 279-0001, Japan; kt900928@gmail.com (K.T.); toshi-s@xj9.so-net.ne.jp (T.S.); 5 Department of Internal Medicine, Navitas Clinic Shinjuku, 4-1-6 Shinjuku 7th Floor of Newoman Bldg, Shinjuku, Tokyo 160-0022, Japan; kami-tky@umin.net (M.K.); hamaki.tamae@gmail.com (T.H.); 6 Department of Internal Medicine, Medical Governance Research Institute, 2-12-13-201 Takanawa, Minato, Tokyo 108-0074, Japan; 7 Department of Gastroenterology, Marru-Clinic Yokosuka, 1-8-7 Yonegahamadori, Yokosuka 238-0011, Kanagawa, Japan; mizunoy3000@gmail.com

**Keywords:** COVID-19, SARS-CoV-2, vaccine, vaccination, Graves’ disease, heart failure, subclinical hyperthyroidism

## Abstract

COVID-19 vaccines are the most critical measure for controlling the COVID-19 pandemic; however, we have little information on their complications. We experienced a case of a patient who developed hyperthyroidism complicated with atrial fibrillation and heart failure on the sixth day after the first dose of COVID-19 vaccination. This case report shows the importance of considering hyperthyroidism as a possible complication after COVID-19 vaccination.

## 1. Introduction

Coronavirus disease 2019 (COVID-19) emerged in Wuhan, Hubei Province, China, in November 2019, and spread to multiple countries, leading to the World Health Organization (WHO) declaring a pandemic on 11 March 2020. According to WHO, as of 23 August 2021, there have been 211 million confirmed cases of COVID-19, including 4.4 million deaths.

Herd immunity through vaccination is essential to control the coronavirus pandemic. Since the BNT162b2 mRNA COVID-19 vaccine developed by Pfizer and BioNTech was approved in the U.S. under Emergency Use Authorization on 11 December 2020 [1], massive vaccination programs using several types of vaccines against COVID-19 are being undertaken around the world. While some variants of severe acute respiratory syndrome coronavirus-2 (SARS-COV-2) such as B.1.617.2 (Delta) impair vaccine-induced protective immune responses, additional booster vaccinations might be helpful for overcoming this problem [2]. According to WHO, as of 22 August 2021, a total of 4619 million vaccine doses have been administered.

Vaccines are a promising measure to control COVID-19; however, there are some problems to be discussed. The most significant is safety. Important complications early after vaccination are inflammatory responses. In a Phase III clinical trial on the BNT162b2 mRNA COVID-19 vaccine [1], 83% of vaccine recipients aged 16 to 55 years developed pain at injection sites, and 47% and 42% of them complained of fatigue and headache, respectively, after the second administration. In the interim analysis of four randomized controlled trials using the ChAdOx1 nCoV-19 vaccine (AZD1222) against SARS-CoV-2 [3], fatigue, headache, feverishness, and myalgia were the most commonly reported systemic adverse reactions. The severity of these inflammatory responses is usually mild to moderate, and these complications resolve spontaneously without sequelae, even if anti-inflammatory and analgesic drugs are sometimes used.

Another concern is immune-mediated adverse events such as myocarditis/pericarditis [4], vaccine-induced immune thrombocytopenia and thrombosis [5], and Guillain–Barré Syndrome [6,7]. These complications are rare, but sometimes cause serious sequelae and can even be fatal [8,9]. Myocarditis/pericarditis occurs at a rate of 12.6 per million, mostly after the second dose of mRNA COVID-19 vaccination in young male adolescents within a few days of vaccination [9,10,11]. Most patients recover spontaneously without significant sequelae, while we cannot deny the possibility of unfortunate outcomes considering cardiac involvements. The situation of vaccine-induced immune thrombocytopenia and thrombosis (VITT) is different from myocarditis/pericarditis. A U.K. research team reported that the mortality of VITT was 22% [12]. Considering the poor prognosis of VITT, the pathogenesis of the disease must be clarified as soon as possible, and appropriate preventive and therapeutic measures must be developed.

Numerous studies on adverse events associated with COVID-19 vaccination have been published; however, they are not yet fully clarified. We experienced a case of a patient who developed hyperthyroidism complicated with atrial fibrillation and heart failure after the first dose of COVID-19 vaccination. A detailed description of this case will provide valuable information on reactions after COVID-19 vaccination.

## 2. Case Presentation

**Figure 1:**
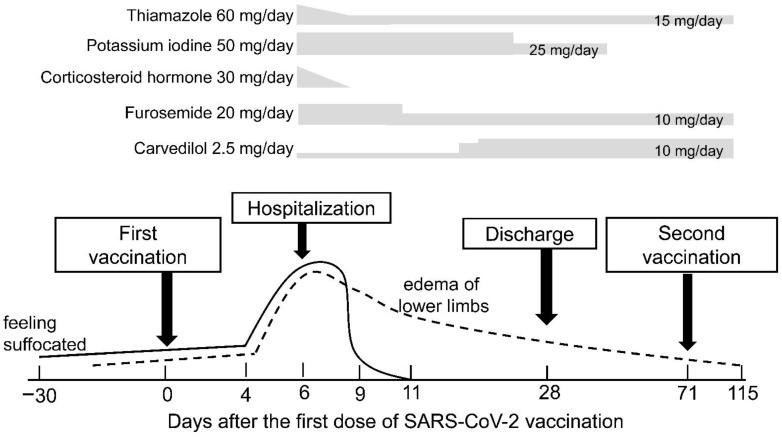
Blood tests on admission showed elevation of serum levels of free triiodothyronine (fT3, 23.2 ng/dL) and free thyroxine (fT4, 3.3 ng/dL) with suppression of thyroid-stimulating hormone (TSH, <0.008 mIU/mL). On Day 23, both fT3 and fT4 were restored to normal levels (3.3 ng/dL and 1.2 ng/dL, respectively). No evidence of recurrent hyperthyroidism was documented after the second vaccination as follows: TSH: <0.008 and 1.029 mIU/mL, fT3 1.9 and 1.9 ng/dL, and fT4 0.73 and 0.59 ng/dL, on Day 80 and 114 post-first vaccination (9 and 43 days after the second), respectively.

A 64-year-old Japanese woman with a medical history of colorectal cancer, diabetes mellitus, and obesity received the first dose of the BNT162b2 mRNA COVID-19 vaccine on April 24 (Day 0) (Figure 1). Abnormal thyroid function had never been documented until COVID-19 vaccination. On Day 1, she developed a low-grade fever, mild fatigue, and pain at the injection site in the left upper arm. These symptoms disappeared the next day (Day 2).

On Day 4, she developed increasing shortness of breath even on a flat road. On Day 6, she visited the emergency room of Tokyo Bay Urayasu Ichikawa Medical Center (Urayasu, Chiba, Japan) due to palpitations, worsening respiratory distress, decreased urine output, edema of both lower legs, and a fever of 38.0 °C. On initial examination, her blood pressure was 161/87 mm Hg. Her pulse rate was 137/min and oxygen saturation was 94% on room air. Her consciousness was clear. Electrocardiogram showed atrial fibrillation, and chest X-ray revealed infiltrations in both lung fields. She was hospitalized immediately after a diagnosis of heart failure complicated with atrial fibrillation was established.

Blood examination after admission showed elevation of serum levels of free triiodothyronine (fT3) 23.2 ng/dL and free thyroxine (fT4) 3.32 ng/dL with suppression of serum levels of thyroid stimulating hormone (TSH) < 0.008 mIU/mL. TSH receptor antibody (TRAb) was positive (33.8 IU/L). Ultrasonography of the thyroid gland revealed the presence of goiter lesions. On color Doppler ultrasonography, there was an increase in vascularization of the parenchyma. The real-time reverse transcription polymerase chain reaction for SARS-Cov-2 testing (nasopharyngeal swab) was negative. She was finally diagnosed as having a thyrotoxic crisis complicated with atrial fibrillation, and heart failure. Acute physiology and chronic health evaluation (APACHE) II score [12] was seven.

With prompt initiation of thiamazole, potassium iodine, corticosteroid, furosemide and carvedilol, her general condition improved rapidly, and her respiratory distress disappeared at Day 11 (five days after admission). Serum levels of thyroid hormones returned to normal on Day 23—fT3 3.27 ng/dL and fT4 1.23 ng/dL. The doctor recommended performing a thyroidectomy. However, the patient refused the surgical treatment option and preferred to be maintained by medication. She was discharged on Day 28.

Considering her underlying conditions, including obesity and diabetes mellitus, she was at a high risk of developing severe COVID-19 pneumonia. She also recognized this risk, and insisted that she would like to receive the second dose of COVID-19 vaccination. After fully explaining the risk of vaccination and obtaining informed consent, we provided her the second dose of the BNT162b2 mRNA COVID-19 vaccine on Day 71. After the second vaccination, she developed a fever of 37.8 °C and pain at the injection site. These adverse events improved spontaneously without the need of medication. No other serious complications developed, and we did not observe any signs of exacerbation of hyperthyroidism. Serum levels of thyroid hormones returned to normal on Day 80—fT3 1.93 ng/dL and fT4 0.73 ng/dL.

As of August 20 (Day 115), her condition is good except for atrial fibrillation and mild edema in both lower extremities. Her current medications are thiamazole (15 mg/day), furosemide (10 mg/day), and edoxaban tosilate hydrate (60 mg/day). She has also taken levothyroxine sodium hydrate (12.5 mg/day) due to hypothyroidism since Day 101. We are now planning catheter ablation for the treatment of persistent atrial fibrillation.

## 3. Discussion

Clinical courses of this patient suggest an association between hyperthyroidism and COVID-19 vaccination. This patient had received regular examination in an outpatient clinic (Navitas Clinic, Shinjuku, Tokyo, Japan) for the treatment of diabetes mellitus and obesity, until she was given the first dose of COVID-19 vaccination, and no signs suggesting hyperthyroidism had been detected prior to the first vaccination. However, ultrasound examination of the neck performed after admission revealed a goiter lesion in the thyroid. These findings suggest the presence of subclinical thyroid disease, which was aggravated after COVID-19 vaccination.

Several factors are known to be associated with exacerbation of hyperthyroidism. These include genetic susceptibility, infection, stress, female, smoking, pregnancy, and drugs such as amiodarone, iodine, or lithium [13,14]. However, this patient did not have any of these risk factors except for being female. Thus, we hypothesized that there was an association between exacerbation of hyperthyroidism and COVID-19 vaccination.

Vaccination is occasionally complicated with autoimmune disorders [15], and several adverse events have been reported after COVID-19 vaccination. These include thrombotic thrombocytopenia [8], myocarditis/pericarditis [16], Guillain–Barré syndrome [6,7], idiopathic thrombocytopenic purpura [17], Graves’ disease [18], and subacute thyroiditis [19]. Interestingly, some complications developed within several days of COVID-19 vaccination [18]. Finsterer reported a case of a patient who experienced exacerbated Guillain–Barré syndrome eight days after vector-based COVID-19 vaccination [6], and Vera-Lastra et al. reported two cases of patients who developed Graves’ disease three days after COVID-19 vaccination [18]. Clinical situations of these cases were comparable to our patient. Given the short interval between vaccination and the onset of autoimmune diseases, it would be reasonable to assume that these patients had mild or subclinical autoimmune diseases that were aggravated by COVID-19 vaccination. 

While the exact mechanisms of these complications following COVID-19 vaccination are not clarified, there are some possibilities to be discussed. It is noteworthy that autoimmune diseases worsened early after COVID-19 vaccination regardless of the vaccine platform, whether mRNA-based or vector-based. Autoimmune/inflammatory syndrome induced by adjuvants is well known as an autoimmune disorder associated with vaccination [20], in which exposure to adjuvants contained in vaccines trigger immune reactions, leading to the development of four conditions: siliconosis, Gulf war syndrome, macrophagic myofasciitis syndrome and post-vaccination phenomena. However, the BNT162b2 mRNA COVID-19 vaccine does not contain adjuvants such as aluminum. Some components of the BNT162b2 mRNA COVID-19 vaccine such as mRNA and lipid nanoparticles might have triggered similar immune responses.

Another possibility is cross-reactivity between the COVID-19 spike protein produced by the BNT162b2 mRNA COVID-19 vaccine and antigens of the thyroid glands [21]. It should be noted that several types of immune-mediated thyroid disorders including autoimmune thyroiditis, subacute thyroiditis and an atypical form of thyroiditis are complications of COVID-19 per se [22] as well as COVID-19 vaccination [18]. These situations are comparable to the findings that myocarditis and idiopathic thrombocytopenic purpura have been reported as complications of COVID-19 [23,24] as well as those of COVID-19 vaccination [16,17]. COVID-19 vaccination elicits immune reactions that are similar to those induced by COVID-19 infection. Considering that thyroid dysfunction is caused by COVID-19, it is not surprising that thyroid dysfunction is also caused by COVID-19 vaccination.

It should be noted that the patient noticed some complaints, such as feeling suffocated and edema of lower limbs, before and at the time of the first vaccine dose. This suggests that the autoimmune process started at least 2–4 weeks before the first dose of COVID-19 vaccination. The patient may have received the first dose of COVID-19 vaccine after the autoimmune process was initiated, possibly causing thyrotoxicosis regardless of the COVID-19 vaccination.

Fortunately, this patient, who had a strong desire for the second dose of the BNT162b2 mRNA COVID-19 vaccine, was able to receive it on Day 71 without any serious complications including recurrence of hyperthyroidism. These findings suggest that the second dose of vaccination is not necessarily contraindicated in patients who have developed hyperthyroidism after the first vaccination. However, the efficacy and safety of the second vaccination for this patient need to be further evaluated. While the Center of Disease Control in the U.S. recommends that the second dose of the Pfizer-BioNTech COVID-19 vaccine should be received 21 days after the first dose of vaccination [25], an optimal interval between the first and the second vaccination remains to be established. This patient received the second dose of vaccine 71 days after the first vaccination. A research team from the U.K. reported that an interval of at least six weeks between the two doses of the Pfizer-BioNTech COVID-19 vaccine increased concentrations of neutralizing antibodies [26]. If expanding the interval between vaccinations strengthens immunity, vaccination with an extended dosing interval may result in stronger adverse reactions. Two analyses of COVID-19 vaccine administration data in the United States showed that 8.6% had delayed the second dose (≤42 days since the first dose) and 3.4% had missed the second dose (>42 days since the first dose) [27]. It is necessary to collect information on the safety of such delayed vaccinations.

The efficacy of the COVID-19 vaccine might decrease as time passes after the second dose of vaccination. Israeli data showed that the rates of confirmed COVID-19 and severe illness were substantially lower among those who received the third (booster) dose of the vaccine [28]. A booster dose of the vaccine has been considered in many countries, and some countries have already started administering a third dose of the vaccine. In this case, the patient has been treated with corticosteroids after the first dose of the vaccine, and the response to the vaccine is unpredictable. We therefore intend to measure the IgG antibodies in the patient’s blood. The patient would be actively encouraged to receive a third dose of the vaccine if antibody levels were found to be low.

In conclusion, this case demonstrates a possibility of exacerbation of subclinical hyperthyroidism after COVID-19 vaccination. Subclinical hyperthyroidism is a common disorder, with a prevalence of 2.1% [29]. When female recipients of COVID-19 vaccines complain of symptoms such as fever, fatigue, and palpitations, we should consider hyperthyroidism as a differential diagnosis. Further studies are warranted to clarify the clinical features, predisposing factors, pathogenesis, prevention and treatment of this complication of COVID-19 vaccines.

## Data Availability

Data sharing is not applicable to this article as no datasets were generated or analyzed during the current study.

## Abbreviations

COVID-19	coronavirus disease 2019
fT3	free triiodothyronine
fT4	free thyroxine
TSH	thyroid-stimulating hormone
TRAb	thyroid-stimulating hormone receptor antibody
TSAb	thyroid-stimulating antibody
VITT	vaccine-induced immune thrombocytopenia and thrombosis
